# Rare Childhood Malignancy

**DOI:** 10.3390/cancers13071504

**Published:** 2021-03-25

**Authors:** Elizabeth M Algar

**Affiliations:** 1 Hudson Institute of Medical Research, Clayton 3168, Australia; elizabeth.algar@monash.edu; 2 Department of Translational Medicine, Monash University, Clayton 3168, Australia

This small collection of six original research papers and two review articles in the Special Issue “Rare Childhood Malignancy” highlights the diversity and importance of empirical research into childhood malignancy, a theme that underpins the significant advances that have been made in treating the diseases that constitute cancers in children. These articles cover significant themes in childhood cancer research and include biological studies on the role of *MYCN* in poor prognosis Wilms’ tumor [1] and the mTOR complexes in rhabdomyosarcoma [2]; studies exploring biologically based treatments in ependymoma [3] and osteosarcoma [4]; a comprehensive description of a novel orthotopic model for radiation-induced glioma which will be invaluable for preclinical testing [5]; and two comprehensive review articles on leukemias. One review describes recently identified rearrangements of *DUX4* with *IGH* in childhood B-cell acute lymphoblastic leukemia (ALL) and the diagnostic challenges associated with identifying the rearrangement [6], and the second proposes that infant acute myeloid leukemia (AML) is a biologically and clinically distinct entity from AML in older children [7]. An additional original research article [8] emphasizes the importance of monitoring for spinal deformities in children with central nervous system (CNS) tumors and the advantages of early assessment and surgical interventions where needed. 

Significant outcomes from selected studies are summarized here. The study by Szemes et al. [1] sheds new light on the role of MYCN in anaplastic Wilms’ tumor. *MYCN* amplification and overexpression is associated with poor outcome in Wilms’ tumor and occurs predominantly in diffuse anaplastic subtypes; however, its impact on Wilms’ tumor biology has not previously been elucidated. The article by Szemes et al. [1] goes some way to addressing this in reporting that the Wilms’ tumor oncogene *LIN28B* is upregulated by MYCN and that the *REST* gene, a suppressor gene associated with germline predisposition to Wilms’ tumor, is repressed by MYCN. Felkai et al. [2] propose triaging rhabdomyosarcoma for intervention with rapamycin based on tumor mTORC1/mTORC2 expression profile as a result of their findings showing a relationship between elevated mTOR expression and a worse response to chemotherapy at relapse, with mTORC2 (rapamycin-insensitive) expression predominant in primary samples. Validation of this study may lead to better directed therapy for rhabdomyosarcoma. Harris et al. [4] report the exciting finding in preclinical models that the second-generation proteasome inhibitor ixazomib is effective in inhibiting osteosarcoma metastases in athymic mice. When metastatic disease is present in osteosarcoma, the prognosis is poor, with only 20–30% survival. Targeting or preventing metastases could lead to significant improvements in disease survival. Finally, Liang et al. [3] show, by deriving differential gene expression from publicly available data for ependymoma, evidence for upregulation of the cell cycle genes *CDK4* and *CCND1*, which was confirmed in primary tumor specimens. Testing the cell cycle inhibitor abemaciclib in a preclinical ependymoma model yielded promising results, suggesting that abemaciclib has potential as a therapeutic for ependymoma, a disease for which more effective treatments are needed.

As childhood cancer is a rare disease, international cooperation, transparency, and resource and data sharing are particularly important in order for key advancements in our understanding of disease biology to be made. Disease biology impacts clinical management, and a united approach, particularly for extremely rare tumors, will ultimately lead to improved outcomes for children with cancer. 
